# Liver Injury Due to Penetration of the Common Iliac Vein by an Extracorporeal Membrane Oxygenation Cannula

**DOI:** 10.7759/cureus.58620

**Published:** 2024-04-19

**Authors:** Naoya Ozawa, Masahiro Konaka, Joji Ito, Masaki Kanzaki

**Affiliations:** 1 Department of General Surgery, Tokyo Bay Urayasu Ichikawa Medical Center, Urayasu, JPN; 2 Department of Intensive Care, Tokyo Bay Urayasu Ichikawa Medical Center, Urayasu, JPN; 3 Department of Cardiovascular Surgery, Tokyo Bay Urayasu Ichikawa Medical Center, Urayasu, JPN

**Keywords:** cannula removal, duodenum injury, liver injury, ascending lumbar vein, penetration, extracorporeal membrane oxygenation support

## Abstract

Extracorporeal membrane oxygenation (ECMO) cannulas inserted through the femoral vein can stray into the ascending lumbar vein. No case has been reported in which the cannula has penetrated the common iliac vein and entered the abdominal cavity. A 52-year-old man was brought to the emergency room with ventricular fibrillation, and the cannula inserted from the left femoral vein for extracorporeal cardiopulmonary resuscitation penetrated the common iliac vein, passed between the pancreas and horizontal portion of the duodenum, and entered the abdominal cavity to reach the hepatic left lateral lobe. The cannula was removed, and organ damage was confirmed through laparotomy. When it is necessary to remove a cannula that has penetrated a vessel, surgical removal is preferable to evaluate the damage and prevent complications associated with removal.

## Introduction

Complications associated with extracorporeal membrane oxygenation (ECMO) include hemorrhage and embolism, whereas complications associated with cannulation include perforation, arterial dissection, and cannula malposition [1-4]. Although previous reports have described an ECMO cannula punctured from the femoral vein straying into the ascending lumbar vein [5,6], injuries to other organs due to intraperitoneal penetration have not been reported to date. There has been a report in which surgical removal of the cannula was considered [5]. However, there are no reports of actual surgical removal of the cannula, and the appropriate removal method has not been established. We encountered a case in which a cannula penetrated the common iliac vein, passed through the retroperitoneal cavity, entered the abdominal cavity, and damaged the liver.

This article was previously presented as a poster at the 76th General Meeting of the Japanese Society of Gastroenterological Surgery on July 7, 2021.

## Case presentation

**Figure 1:**
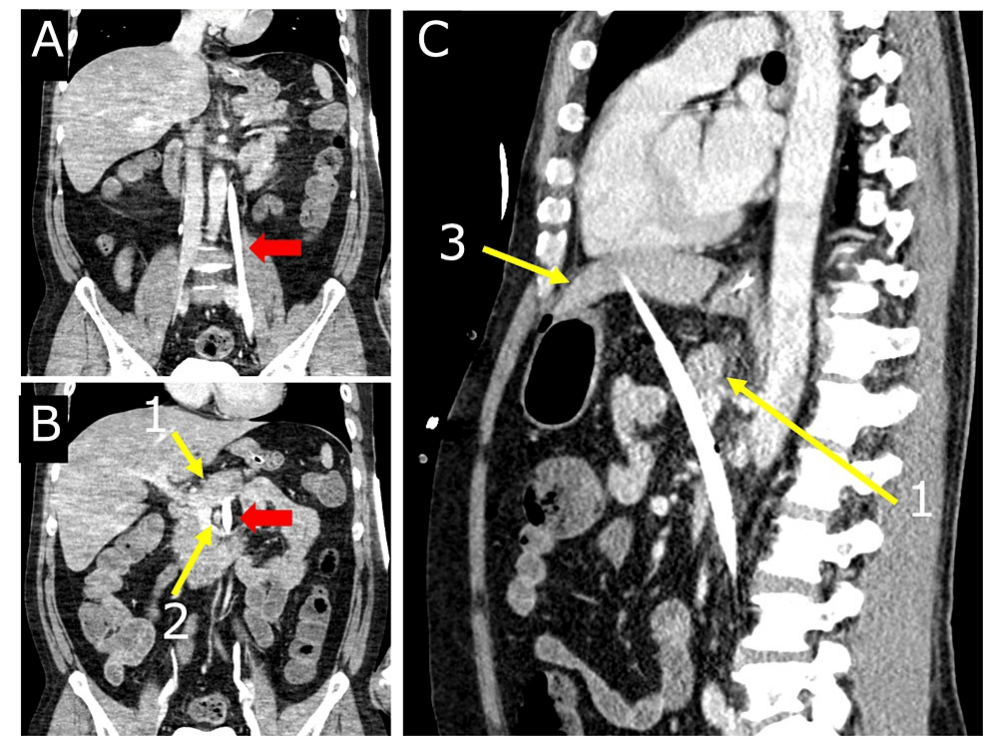
Contrast-enhanced CT (A) The venous cannula (red arrow) had strayed into the left ascending lumbar vein and perforated the retroperitoneal space. (B) The venous cannula (red arrow) had passed between the pancreas and the horizontal portion of the duodenum. (C) The tip of the venous cannula appeared to be impaled in the hepatic left lateral lobe. 1: Pancreas, 2: Superior mesenteric artery, 3: Liver

A 52-year-old man with no known medical history was found to have collapsed and was brought to the emergency room with ventricular fibrillation. Extracorporeal cardiopulmonary resuscitation (ECPR) was performed, and cannulation was performed through the left femoral artery and vein using a percutaneous cardiopulmonary assist device. The position of the guidewire was confirmed by a simple radiograph, and the arterial cannula was successfully implanted. No reverse bleeding was observed from the venous cannula. The patient’s heartbeat resumed during the puncture procedure. Emergency cardiac catheterization was performed by stenting a stenotic lesion in the anterior descending branch of the left coronary artery. A subsequent contrast-enhanced computed tomography (CT) scan showed that the venous cannula penetrated the common iliac vein, passed through the retroperitoneal cavity, between the pancreas and the horizontal portion of the duodenum, and entered the abdominal cavity, reaching the hepatic left lateral lobe at its tip (Figure 1). An emergency laparotomy was performed for suspected pancreaticoduodenal, common iliac vein, and left lateral lobe injuries.

**Figure 2:**
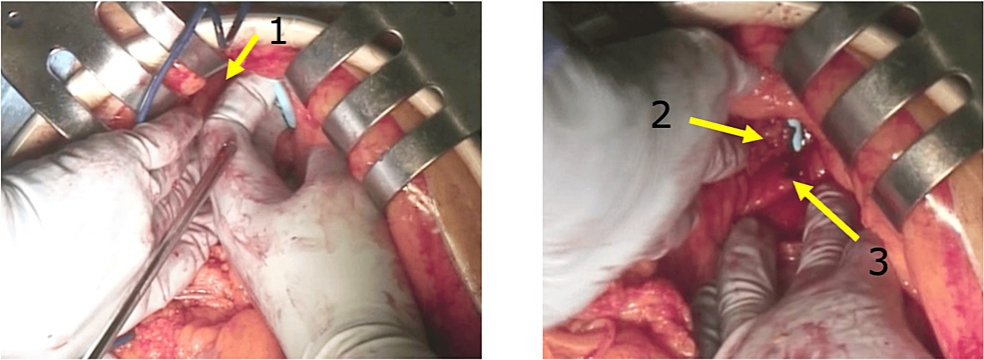
Intraoperative findings (A) The cannula tip penetrated the liver, causing liver injury. (B) The cannula passed between the pancreas and duodenum. 1: Liver, 2: Pancreas, 3: Duodenum

Injuries to the hepatic left lateral lobe and pancreatic injury were observed (Figure 2). The bleeding from the liver was stopped by compression. A duodenal injury was ruled out after the horizontal and ascending legs of the duodenum and proximal jejunum were mobilized to expose the venous cannula completely. Although hemostasis was achieved by compression of the injured liver and pancreas, a TachoSil^®^ tissue sealing sheet (TachoSil, CSL Behring, Pennsylvania, US) was used due to the risk of bleeding from subsequent antiplatelet therapy and hypothermia. Finally, the venous cannula was removed, and the left common iliac vein was repaired using the TachoSil® tissue sealing sheet. The bleeding volume was 250 mL. Postoperative blood test results were aspartate transaminase (AST) 143 U/L, alanine transaminase (ALT) 159 U/L, total bilirubin 0.61 mg/dL, and amylase 138 U/L. There were no postoperative complications, including pancreatitis. Hypoxic encephalopathy due to cardiac arrest was observed, and the patient was transferred to another hospital 35 days after the surgery.

## Discussion

This case demonstrated two points: the ECMO cannula can penetrate the abdominal cavity, and organ damage should be assessed and removed during surgery to prevent complications.

First, ECMO cannulas can also penetrate the abdominal cavity. Vascular complications have been reported in 10% of adults who underwent an introduction of ECMO through the femoral artery, including acute embolism/thrombosis, dissection of the femoral artery, pseudoaneurysm, inguinal hematoma, femoral artery perforation, and compartment syndrome [3]. To date, several cases of ECMO cannulas straying into the ascending lumbar vein have been reported [5,6]. Generally, cannulas inserted into the left femoral vein are more likely to enter the ascending lumbar vein than those inserted into the right femoral vein. This is because the left ascending lumbar vein flows more linearly into the common iliac vein than the right ascending lumbar vein, and the left ascending lumbar vein develops as a collateral circulation because the left common iliac vein is compressed by the right common iliac artery and vertebral body, which is more likely to develop than on the right side (May-Thurner syndrome) [7-9]. In this patient, the catheter may have penetrated the common iliac vein by straying into the ascending lumbar vein, possibly as a result of being inserted via the left femoral vein. The cannula then penetrated the abdominal cavity via the retroperitoneal cavity and omental bursa. Here, the cannula passed through the sparse tissue, suggesting little organ damage relative to the length of the cannula that passed through. This is the first case of intraperitoneal penetration that we have found in the literature.

Second, surgical removal is preferable for assessing damage and preventing complications associated with removal when it is necessary to remove a cannula that has strayed into unintended vessels or penetrated outside the vessel. In some previous cases, the cannula strayed into the ascending lumbar vein without causing problems with blood removal, suggesting that negative pressure for blood removal may not reveal hemorrhagic complications [6]. Although one report described a venous cannula straying into the ascending lumbar vein and its removal under X-ray fluoroscopic guidance after confirming minimal extravascular leakage of contrast medium [5], there is no report of surgical removal of the cannula found to date. In this case, it was confirmed by laparotomy that there was no damage to the duodenum, and the liver injury was hemostatically controlled. However, if the cannula had been removed without surgery, there would have been a risk of peritonitis, intra-abdominal bleeding, or retroperitoneal hematoma.

Since anticoagulants are used during ECMO and hypothermia therapy is also used in ECPR, surgical removal of the cannula may be preferable if the cannula penetrated the vessel or if there is suspicion of organ damage, as it allows for the evaluation of vascular or organ injuries and may prevent complications.

It is preferable to perform a puncture of the common iliac vein from the right side rather than the left. We found three case reports of ECMO cannulas straying into the ascending lumbar vein, including one case in which the catheter entered the left ascending lumbar vein after puncturing the left femoral vein and two cases in which the catheter entered the right ascending lumbar vein after puncturing the right femoral vein. Morita et al. reported that the femoral vein catheter strayed into the left ascending lumbar vein in five (17.2%) of the 29 cases in which the left femoral vein was punctured, which was significantly more frequent (p < 0.05) than in the right ascending lumbar vein in one (0.9%) of the 109 cases in which the right side was punctured [7]. In this case, the cannula may have strayed into the left ascending lumbar vein after it was inserted into the left femoral vein; therefore, it may be better to insert an ECMO cannula into the right femoral vein.

## Conclusions

We present a case in which an ECMO cannula, inserted from the left femoral vein, penetrated the common iliac vein, and entered the abdominal cavity, causing liver injury. This case demonstrates that in situations where it is necessary to remove a cannula that has penetrated a vessel or entered the abdominal cavity, surgical removal is preferable to assess the organ damage and prevent complications associated with its removal such as retroperitoneal hematoma or intraperitoneal bleeding.
